# Influenza A H1N1 Induced Disturbance of the Respiratory and Fecal Microbiome of German Landrace Pigs – a Multi-Omics Characterization

**DOI:** 10.1128/Spectrum.00182-21

**Published:** 2021-10-06

**Authors:** Laurin Christopher Gierse, Alexander Meene, Daniel Schultz, Theresa Schwaiger, Charlotte Schröder, Pierre Mücke, Daniela Zühlke, Tjorven Hinzke, Haitao Wang, Karen Methling, Bernd Kreikemeyer, Jörg Bernhardt, Dörte Becher, Thomas C. Mettenleiter, Michael Lalk, Tim Urich, Katharina Riedel

**Affiliations:** a Institute of Microbiology, University of Greifswald, Greifswald, Germany; b Institute of Biochemistry, University of Greifswald, Greifswald, Germany; c Friedrich-Loeffler-Institut, Greifswald-Insel Riems, Greifswald, Germany; d Institute for Medical Microbiology, Virology and Hygiene, Rostock University Medical Centre, Rostock, Germany; e Institute of Marine Biotechnology e.V., Greifswald, Germany; University of Nebraska-Lincoln

**Keywords:** 16S rRNA gene sequencing, influenza A, microbiome, biomedical model swine, metabolomics, metaproteomics, multi-omics

## Abstract

Seasonal influenza outbreaks represent a large burden for the health care system as well as the economy. While the role of the microbiome has been elucidated in the context of various diseases, the impact of respiratory viral infections on the human microbiome is largely unknown. In this study, swine was used as an animal model to characterize the temporal dynamics of the respiratory and gastrointestinal microbiome in response to an influenza A virus (IAV) infection. A multi-omics approach was applied on fecal samples to identify alterations in microbiome composition and function during IAV infection. We observed significantly altered microbial richness and diversity in the gastrointestinal microbiome after IAV infection. In particular, increased abundances of *Prevotellaceae* were detected, while *Clostridiaceae* and *Lachnospiraceae* decreased. Moreover, our metaproteomics data indicated that the functional composition of the microbiome was heavily affected by the influenza infection. For instance, we identified decreased amounts of flagellin, correlating with reduced abundances of *Lachnospiraceae* and *Clostridiaceae*, possibly indicating involvement of a direct immune response toward flagellated *Clostridia* during IAV infection. Furthermore, enzymes involved in short-chain fatty acid (SCFA) synthesis were identified in higher abundances, while metabolome analyses revealed rather stable concentrations of SCFAs. In addition, 16S rRNA gene sequencing was used to characterize effects on the composition and natural development of the upper respiratory tract microbiome. Our results showed that IAV infection resulted in significant changes in the abundance of *Moraxellaceae* and *Pasteurellaceae* in the upper respiratory tract. Surprisingly, temporal development of the respiratory microbiome structure was not affected.

**IMPORTANCE** Here, we used swine as a biomedical model to elucidate the impact of influenza A H1N1 infection on structure and function of the respiratory and gastrointestinal tract microbiome by employing a multi-omics analytical approach. To our knowledge, this is the first study to investigate the temporal development of the porcine microbiome and to provide insights into the functional capacity of the gastrointestinal microbiome during influenza A virus infection.

## INTRODUCTION

Influenza epidemics occur with seasonal characteristics, predominantly in cold months of temperate climates, causing substantial morbidity and mortality. Each year, an estimated 1 billion cases of influenza occur globally, including 3 to 5 million severe cases resulting in 300,000 to 600,000 influenza-associated fatalities (1). Particularly, children (0 to 4 years), people with chronic diseases, and the elderly (>65 years) bear a great risk of severe disease (2, 3). The total per capita costs of influenza cases were calculated to range from $27 to $52 in Europe and from $45 to $63 in the United States (4). In 2015, the U.S. health care system and society were burdened with an estimated $11.2 billion in direct and indirect costs (e.g., medical costs or reduced productivity caused by absence from work) (5, 6). The impact of seasonal influenza on the population and the health care system is particularly dramatic in middle- to low-income countries. For example, in 2002 the case-fatality ratio during an influenza outbreak in Madagascar was 2.5%, which was similar to the estimated case-fatality ratio (2 to 3%) of the 1918 to 1919 pandemic (7).

Swine represent a particularly good pathogenicity model for influenza A virus (IAV) research and vaccine development because they are genetically and physiologically closely related to humans. Furthermore, clinical symptoms and pathology upon IAV infection in swine resemble those of humans (8). Moreover, the IAV receptors in human and porcine airways show a similar distribution (9, 10). Most importantly, swine is a natural host for IAV, the primary cause of respiratory diseases in pigs, which is a great economic burden to farmers (8). The susceptibility of swine to both avian and human IAVs makes it a “mixing vessel” for novel influenza virus strains by genetic reassortment between avian and human viruses (11, 12). Transmission of IAV between humans and swine occurs sporadically (13) and can be seen as a dead-end zoonotic event, while the reverse-transmission is relatively frequent and causes the large diversity of IAVs in swine (14). A prominent example for a widespread human pandemic infection with a novel swine-origin IAV is the H1N1pdm2009 pandemic in the beginning of 2009 (15). This virus, which was never detected in pigs before, carried a unique reassorted composition of genes related to North American and Eurasian H1N1 swine viruses (16). Beyond that, the pandemic IAV H1N1pdm2009 was characterized by a high infection and transmission efficiency in pigs, with clinical disease and viral replication similar to those of endemic influenza strains (17, 18).

In the past decade, several studies addressed the composition of the porcine gastrointestinal microbiome (19–23). In addition, one meta-analysis compared more than 80 studies to define core microbiota of the porcine gut (24). However, only few studies investigated the impact of respiratory infections, like influenza, on the host microbiome, and almost all of them analyzed the murine model (25–30), while studies in the porcine model were completely missing. Moreover, most murine studies were based on the characterization of the microbiome using 16S rRNA gene sequencing data, leaving the functional characterization of the microbiome largely unexplored. In the collaborative and interdisciplinary project “KoInfekt,” we are currently investigating how monocausal IAV infections and bactoviral coinfections affect host fitness, health status, immune response, and microbiome, employing swine as a model (31–35). For instance, Schwaiger and colleagues analyzed the systemic as well as the local immune response of the same pigs as those used in this study (31). In comparison to the healthy cohort, the infected pigs did not show any differences in animal performance (e.g., weight gain) or any clinical symptoms. Interestingly, influenza A virus matrix protein was detectable only at day 4 after the first infection in the nose, trachea, und lung but was absent after the second infection at day 21, pointing toward a fast recovery. The highest inflammation scores in nose and lung were detected at day 7, constantly decreasing to the end of the experiment. Immune response mirrored by decreased numbers of peripheral blood lymphocytes and an increase of infiltrating leukocytes in the lung was observed. Further, enhanced perforin expression in αβ and γδ T cells in the respiratory tract indicated a cytotoxic T cell response restricted to the route of virus entry. Moreover, increasing frequencies of CD8αα-expressing αβ T cells were observed after the first viral infection, possibly inhibiting uncontrolled inflammation in the respiratory tract (31).

Another central goal of the consortium was to evaluate the longitudinal influence of respiratory infections on the porcine upper respiratory and gastrointestinal microbiome. Therefore, this study aimed to characterize the impact of an H1N1 infection on structure and function of the porcine upper respiratory and gastrointestinal microbiome over 25 days and to identify possible indicators specific for influenza virus infection. To this end, we applied an integrated multi-omics approach consisting of 16S rRNA gene sequencing, metaproteomics, and metabolomics on fecal samples and performed 16S rRNA gene sequencing on nasal swabs from influenza A H1N1-infected swine. Knowledge gained on the effect of IAV infection on the respiratory and gastrointestinal microbiome points toward disturbance of the taxonomic as well as the functional composition of the porcine microbiome and will later serve as a reference data set for bactoviral coinfections.

## RESULTS AND DISCUSSION

### Respiratory tract microbiome.

**FIG 1:**
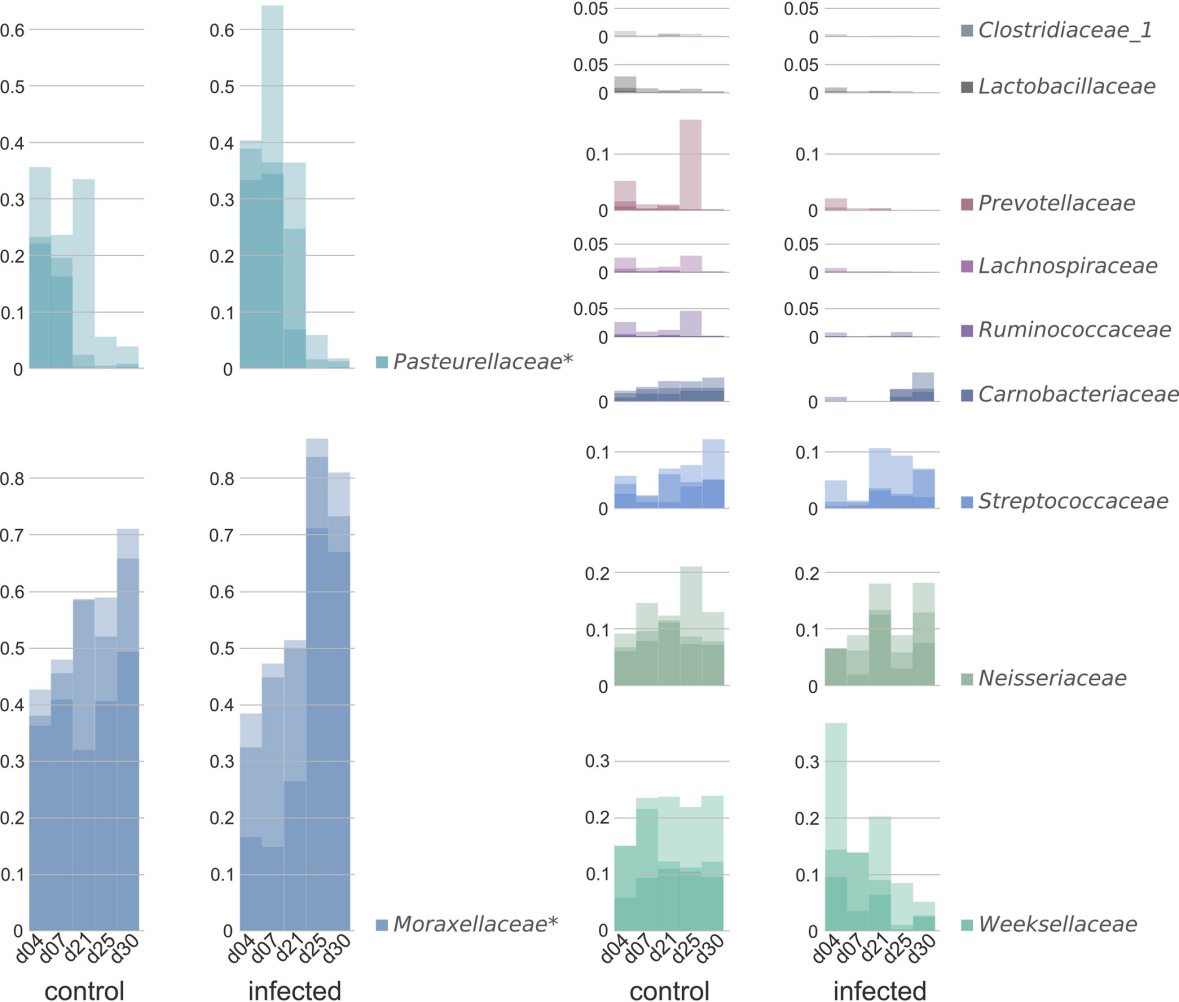
Composition and development over a time period of 30 days of the respiratory microbiome of 8-week-old pigs under healthy conditions (*n* = 3) and after IAV H1N1 infection (*n* = 3) based on analysis of 16S rRNA genes from nasal swabs. Animals were first infected with IAV H1N1 at day 0, followed by a second infection on day 21. Significant changes in the temporal development were marked with an asterisk. Bars are shown as an overlay and represent the three swine at the corresponding sampling day, resulting in a darker shade, by higher accordance between the individual animals. For better illustration, only the 11 most abundant families were shown. *x* axes: sampling day; *y* axes: relative abundance.

**IAV infection affects composition of the upper respiratory tract microbiome.** Due to the limited biomass obtained from the nasal swabs, characterization of the upper respiratory tract (URT) microbiome prior to and during influenza A H1N1pdm09 infection was not possible by metaproteome analyses but was performed exclusively by 16S rRNA gene sequencing. Sequencing revealed 147 (±54) unique bacterial and archaeal amplicon sequence variants (ASVs) in the healthy, and 90 (±18) ASVs in the infected, URT microbiomes of swine. The obtained numbers of bacterial and archaeal ASVs, as well as standard deviation (SD) and Shannon and Simpson index, are summarized in Table 1. Welsh *t* test (*P* = 0.05) revealed a significantly reduced number of ASVs in the infected animals compared to that in the healthy control pigs. Further, microbial richness as well as the richness dynamics was significantly lower in the infected animals, according to Shannon (*P* = 0.005) and Simpson index (*P* = 0.002). In contrast, a study in the murine model demonstrated that the microbial diversity in the URT was unaffected by IAV infection (29). These differences could be explained by the use of a different animal model and the distinctive sampling method (nasal swab versus whole organ homogenate). Moreover, independent of the health status, high interindividual variation within the URT microbiome of the pigs was observed. As it is known that the development of the URT microbiome can be affected by several factors (e.g., the mother, feeding, weaning, delivery mode, environmental and housing conditions, vaccination, and antimicrobial exposure), this individual variation was not surprising (36–39). Furthermore, host genetics were considered to have minor effects on the composition of URT microbiome, in contrast to the sputum microbiome, which is influenced equally by host genetics and the environment (40). Nevertheless, comparing the URT microbiomes under healthy and infection conditions revealed significant (analysis of variance [ANOVA], *P* < 0.05) differences in the taxonomic composition. 16S rRNA genes were assigned mainly to the phyla *Proteobacteria*, *Bacteroidetes*, and *Firmicutes* (Table S1). Further, *Moraxellaceae*, *Pasteurellaceae*, *Weeksellaceae*, and *Neisseriaceae* were identified as predominant under both conditions (Fig. 1). These findings were in good accordance with a comparable study of Espinosa-Gongora et al. analyzing the nasal microbiome of Staphylococcus aureus-carrying and noncarrying pigs and identifying *Proteobacteria* as the dominant phylum, including the abundant occurrence of members of the families *Moraxellaceae* and *Pasteurellaceae* (41). Analysis of the URT microbiome revealed a few significant (ANOVA, *P* = 0.05) infection-induced shifts in the composition of the respiratory microbiome on the phylum level (Fig. S1) as well as on the family level. *Pasteurellaceae* (*P* = 0.02) were increased at early time points on day 4 and day 7 followed by a constant decrease, like the temporal development under healthy condition. Furthermore, *Moraxellaceae* (*P* = 0.00006) were detected in lower relative abundance in the microbiome of the infected animals at days 4 and 7, followed by a strongly increased abundance at days 25 and 30 after infection. Although the relative abundance of *Pasteurellaceae* and *Moraxellaceae* was significantly higher during IAV infection, consistently increased relative abundance of *Moraxellaceae* and decreased abundance of *Pasteurellaceae* were observed over the period of 30 days under both conditions. Moreover, a constant decrease of the family *Weeksellaceae* from day 4 to day 30 after infection, as well as a slightly decreased abundance of *Neisseriaceae*, was detected (Fig. 1). Infection-induced alterations in the microbiome structure were expected, as it is well known that the human respiratory microbiome, which is stable under healthy conditions (42), can be affected by viral infections (43). For instance, changes in the relative abundance of the genera *Staphylococcus* and *Bacteroides* after live attenuated influenza vaccination (44), as well as increased abundances of *Haemophilus* and *Fusobacterium* in the URT of humans in response to severe influenza, have been described (45). In good accordance with our findings of significantly altered microbial composition due to influenza A infection, Traxler et al. (33) detected significant differences in the composition of breath volatile organic compounds (VOCs) produced by bacteria. Comparison of the VOC profiles from healthy and IAV-infected swine revealed increased levels of acetaldehyde, propanal, N-propyl acetate, methyl methacrylate, styrene, and 1,1-dipropoxypropane on day 4 in the infected animals. This observation could be linked to disease progression, as the compounds correlated with the detection of the viral matrix protein (33).

**TABLE 1:** Alpha-diversity parameters of the respiratory tract microbiome from healthy and IAV H1N1-infected swine over 30 days, including the average number of ASVs, standard derivation (SD), Shannon index, and Simpson index

Alpha-diversity parameter	Value for:									
	Control animals					Infected animals				
	d04	d07	d21	d25	d30	d04	d07	d21	d25	d31
ASV	187	111	136	216	85	113	83	98	67	89
SD	66	16	31	137	22	35	13	23	15	7
Shannon	2.35	2.03	2.23	2.36	1.91	1.97	1.69	1.94	1.68	1.64
Simpson	0.83	0.82	0.82	0.80	0.75	0.78	0.71	0.80	0.66	0.66

**FIG 2:**
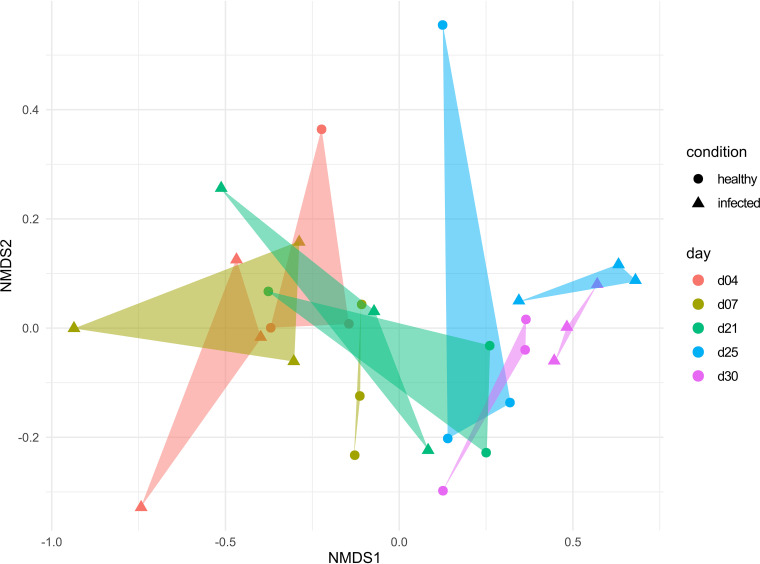
Nonmetric multidimensional scaling (NMDS) plot, based on Bray-Curtis dissimilarities of the taxonomic 16S rRNA gene profile from nasal swabs of pigs over 30 days after IAV H1N1 infection.

A nonmetric multidimensional scaling (NMDS) analysis was performed to test whether the viral infection influenced the time-dependent development of the respiratory tract microbiome (Fig. 2). We observed a unilateral time-dependent succession in the natural development of the healthy URT microbiome, characterized by a distribution of the clusters along the NMDS1-axis from left to right. Further, our results showed that this temporal succession of the nasal microbiome structure was almost unaffected by the IAV infection. The observation that the IAV infection did not affect the natural development was surprising, as we detected significant changes within the relative abundance of several dominant members of the microbiome and decreased microbial richness. Using animals from the same trial, Schwaiger and colleagues analyzed the porcine immune response to the IAV H1N1pdm09 infection and described only mild inflammatory changes in the nasal mucosa, trachea, and lung, which were decreasing until day 25 after the first infection (31). The H1N1pdm09-infected animals showed mild focal, necrotizing rhinitis with loss of epithelial cells and IAV matrix protein-positive respiratory epithelial cells within the lesions at day 4. The IAV matrix protein was detectable only at day 4 after the first viral infection in the nose, trachea, and lung. The highest inflammation score was detected at day 7 in the nose and lung of the pigs, which then decreased slightly and remained constant until the end of the experiment. Since the second viral infection produced no clinical symptoms in the upper respiratory tract and the viral matrix protein was not detected (31), one could speculate that the sublethal infection did not lead to long-term colonization of the IAV in the upper respiratory tract, and due to this viral cleanup/loss, low inflammation scores, mild symptoms, and only minor alterations within the temporal succession of the respiratory microbiome were observed.

For further studies of the respiratory microbiome, the use of bronchoalveolar lavages (BALs) instead of nasal swabs might be beneficial, as the biomass obtained from nasal swabs is limited. The use of BALs could pave the way to perform multi-omics on the respiratory microbiome and potentially identify metabolic pathways involved in the alterations of the VOC profiles.

### Gastrointestinal tract microbiome.

**General multi-omics results.** By employing an integrated multi-omics approach consisting of 16S rRNA gene sequencing, metaproteomics, and metabolomics on fecal samples from healthy and infected swine, structure and function of the gastrointestinal microbiome were elucidated prior to and during sublethal influenza A H1N1pdm09 infection. Subsequently, the structural and functional composition of the gut microbiome was compared to a reanalyzed data set of the healthy porcine microbiome from the same experiment (35). 16S rRNA gene sequencing identified 912 (±88) ASVs from feces of the IAV-infected animals, which is in good accordance to the ASV numbers detected for the gastrointestinal microbiome of healthy swine (35). According to Shannon index, infected animals had a significantly increased (Welsh *t* test, *P* < 0.05) microbiome diversity (*P* = 0.02) (Table S2A and B). This is in contrast to the microbial richness of the URT microbiome, which was significantly reduced after the IAV infection (Table 1). The increased microbial richness in the gastrointestinal tract might be an indicator for a systemic effect of the viral infection, as described in mice (26). Using metaproteomics, we identified a mean of 4,255 (±364) protein groups (PGs) under healthy conditions and 4,168 (±604) PGs after IAV H1N1 infection. These values are in good accordance with our previous study of the healthy fecal microbiome and underline the reproducibility of the previously established multi-omics pipeline (35). The total number of identified PGs from the individual samples is depicted in Table S3A and B. The metabolome analysis focused on short-chain fatty acids (SCFAs). Using nuclear magnetic resonance (NMR) analysis, we quantified the amounts of SCFAs of fecal samples during the infection experiment (Table S4) and compared the results to those of our previous study on the healthy gastrointestinal microbiome (35) and other comparable studies (46–48).

### Influence of IAV H1N1 infection on the taxonomic composition of the gastrointestinal microbiome.

**FIG 3:**
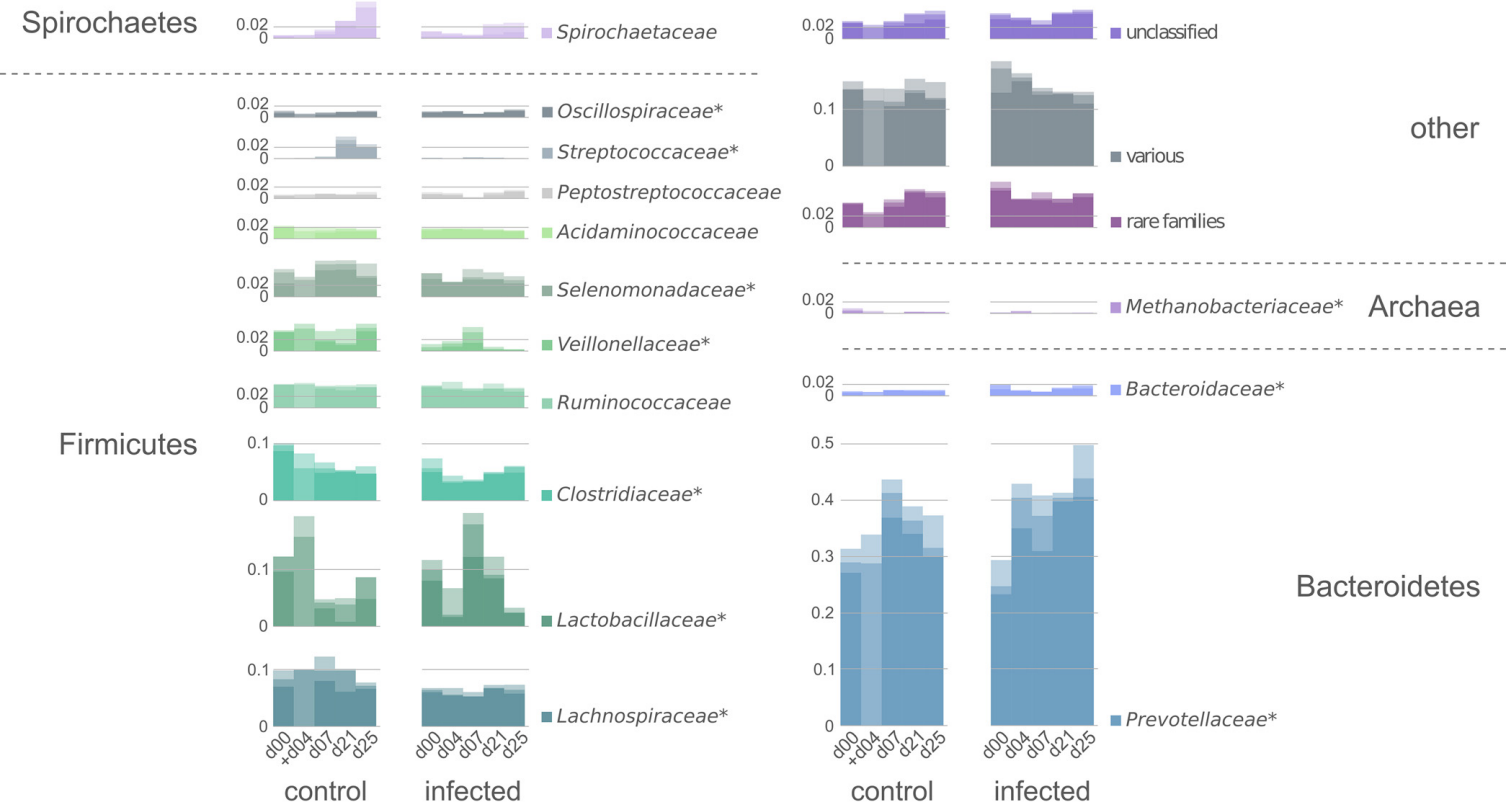
Metaproteome-based taxonomic composition, on the family level, of the microbiome from fecal samples of healthy (*n* = 3) and IAV H1N1-infected (*n* = 3) pigs over a time period of 25 days shown in relative abundance. Significant changes (ANOVA, *P* value = 0.05) in the relative abundance (increase or decrease) driven by the infection are highlighted with an asterisk (*) behind the corresponding family. Bars are shown as an overlay and represent the three swine at the corresponding sampling day, resulting in a darker shade, by higher accordance between the individual animals. *x* axes: sampling day; *y* axes: relative abundance; +: one sample missing for the corresponding day.

Taxonomic profiles of the fecal microbiome were investigated by 16S rRNA gene sequencing and metaproteome analysis. Despite the use of different taxonomic databases (16S rRNA gene sequencing: SILVA; metaproteomics: NCBI), Pearson correlation of 0.76 reveals good accordance between both taxonomic profiling approaches, as both omics techniques identified similar proportions of the dominant families of the gastrointestinal tract microbiome (e.g., *Prevotellaceae*, *Lachnospiraceae*, *Ruminococcaceae*, *Clostridiaceae*, and *Lactobacillaceae*) (Fig. S2). Further, both omics approaches identified altered temporal development of the family *Lactobacillaceae* and a decreased amount of *Streptococcaceae* during IAV infection at the end of the experiment. High congruency between 16S rRNA gene sequencing and metaproteomic data was observed in a previous study investigating healthy pigs (35). In the following text, all taxonomic data on the microbial composition of the gastrointestinal microbiome will be presented based on metaproteomic data. The complementary 16S rRNA gene sequencing profiles are provided in the appendix (Fig. S2; Table S5). Independent of the health status of the animals, the largest proportion of identified PGs were of bacterial origin (74 to 89%), followed by eukaryotic (9 to 20%), various (0.06 to 0.1%), unclassified (0 to 0.0002%), archaeal (0 to 0.01%), and viral (0 to 0.0006%) PGs. The majority of identified bacterial PGs could be assigned to the phyla *Firmicutes* and *Bacteroidetes* (Table S3). After the H1N1 infection, we detected an altered ratio of *Firmicutes* and *Bacteroidetes*. In particular, we observed a decrease in relative abundance of the phylum *Firmicutes* from an average of 0.47 to 0.38, while the *Bacteroidetes* increased slightly from 0.33 to 0.38 on average. Similar to our findings, Zhu and colleagues observed that the *Firmicutes*/*Bacteroidetes* ratio of the murine gastrointestinal tract microbiome was affected by a hepatitis B virus infection (49). ANOVA (*P* value = 0.05) revealed that the IAV infection also induced significant alterations of the composition of the gastrointestinal tract microbiome on the family level. In particular, significant changes were detected in the relative abundance of the families *Lachnospiraceae* (false-discovery rate [FDR] = 0.0003), *Clostridiaceae* (FDR = 0.0003), *Veillonellaceae* (FDR = 0.00003), *Streptococcaceae* (FDR = 0.00000007), and *Selenomonadaceae* (FDR = 0.015) but also in that of the families *Prevotellaceae* (FDR = 0.004) and *Bacteroidaceae* (FDR = 0.00008) (Fig. 3). According to ANOVA, the relative abundance of the most abundant family, *Prevotellaceae*, was significantly increased in the microbiome of IAV-infected swine compared to that in the healthy cohort (Fig. 3). Higher relative abundances of *Prevotellaceae* were linked to probiotic effects in the gut of weaned pigs, resulting in higher levels of luminal IgA, which support the maintenance of a tolerant noninflammatory host-microbial relationship (23, 50). Further, we tested for significant changes in relative abundance at the individual sampling days. Notably, the temporal development of *Lactobacillaceae* was significantly altered in response to the IAV infection. In the healthy microbiome, a strong increase in relative abundance of the *Lactobacillaceae* on day 4, followed by a drastic decrease to day 7, with a subsequent stabilization at this level until day 25, was observed (Fig. 3). In contrast, a significantly decreased relative abundance on day 4 (FDR = 0.01) in the microbiome of IAV-infected animals, followed by strong increased abundance on day 7, was detected compared to that of the control group. A detailed table of significantly altered families is provided in the supplemental material (Table S6). The development of the porcine microbiome can be affected by several factors, such as diet composition, genetics, environment, antibiotic exposure, or infections (37, 51–54). However, knowledge on the impact of influenza virus infections on the porcine gastrointestinal tract microbiome is still scarce.

**FIG 4:**
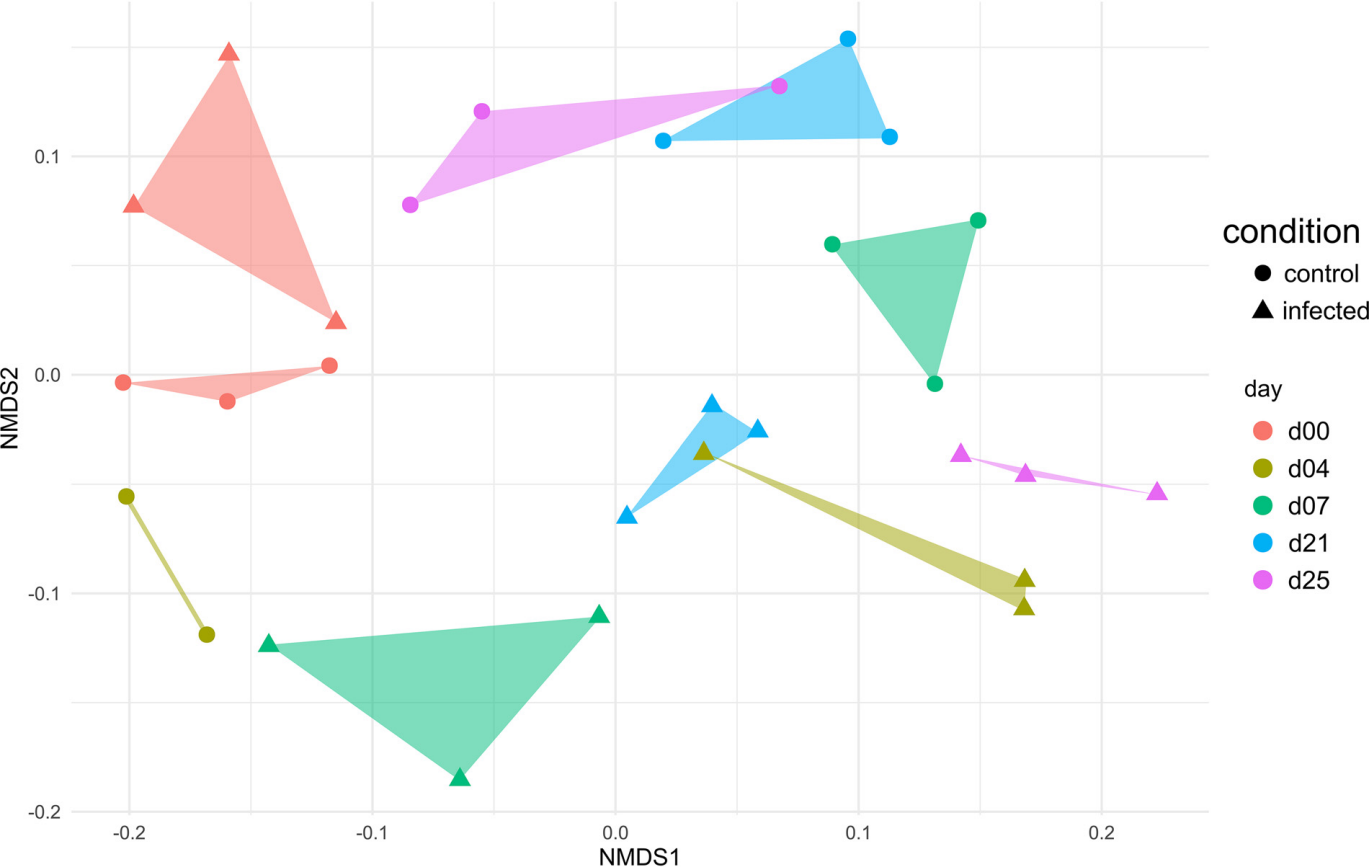
NMDS plot, based on Bray-Curtis dissimilarities of the taxonomic profile (metaproteome) from the fecal microbiome of healthy and influenza A H1N1-infected pigs over a period of 25 days.

Therefore, NMDS analysis was performed to unravel temporal dynamics of the gastrointestinal tract microbiome in response to the IAV infection. As expected, clusters of the healthy and IAV-infected animals were separated according to the sampling day, indicating a relatively small interindividual variance as described previously (35). While the clusters were located close together at day 0 of the experiment, they deviated from each other during the infection (Fig. 4), enabling a differentiation between healthy and infected animals on NMDS axis 2. This finding was also supported by the NMDS plot of the 16S rRNA gene sequencing data (Fig. S3). Further, we observed a development in composition back to day 0 in the healthy microbiome, while after viral infection, the clusters of the later time points did not rearrange to day 0 composition. Based on our results, we propose that the gastrointestinal microbiome is disturbed during IAV infection. This has also been described in the murine model (26). To further support our findings, a hierarchically clustered heatmap (Fig. S4) was constructed using MeV (55). The results confirmed separated branches of the hierarchical tree related to the health status (healthy or IAV-infected) of the animals (Fig. S4).

### IAV induced disturbance in the functional composition of the gastrointestinal microbiome.

**FIG 5:**
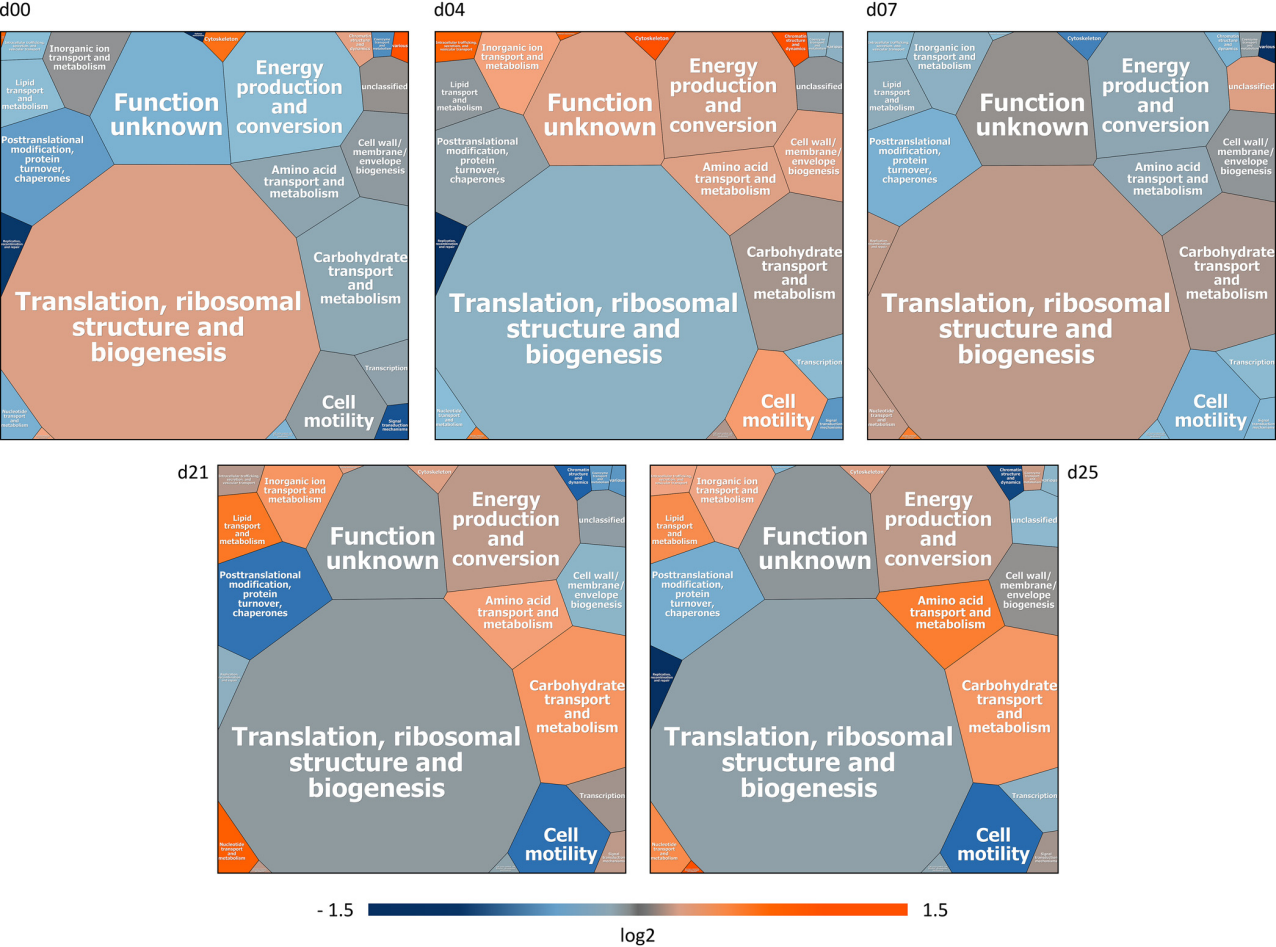
Voronoi Treemaps highlighting changes in the functional composition of the porcine gastrointestinal microbiome, based in the eggNOG categories, during IAV infection. Data of the IAV-infected swine were compared to healthy control animals previously published by Gierse et al. (35). The size of the fields represents the average abundance of the category for all conditions and time points. For comparison, the log_2_ fold change (infected/healthy) of the corresponding sampling days was used. Orange fields represent a 2.8-fold increase (log_2_fc value of 1.5), dark blue fields represent a 0.35-fold decrease (log_2_fc value of −1.5), and gray fields represent no change from comparing the functional assignment of identified PGs out of fecal samples from infected and healthy animals at the corresponding sampling days.

Our comparative metaproteome analysis of the gastrointestinal microbiomes from healthy and IAV-infected pigs revealed that independent of the condition, the identified PGs were similarly classified among the categories of the eggNOG database (Fig. S5). Notably, 89% of all identified PGs could be assigned to specific biological functions, while 11% of the PGs were not assigned to functions according to the classification using the eggNOG database. Most PGs were assigned to the category “translation, ribosomal structure and biogenesis,” showing similar proportions of highly abundant proteins, like proteins from the large and small ribosomal subunits. The second-most abundant class of PGs was assigned to the category “energy production and conversion,” including proteins involved in ATP production (ATP-synthase complex) or electron transport (e.g., rubrerythrin). While the numbers of PGs assigned to these categories were found to be relatively stable in healthy animals, higher variations were observed between the sampling days in the infected animals (Table 2; Table S7). In contrast to the functional assignment of the healthy microbiome, which was rather robust between different sections (56), unaffected by different diets (57), and reported to maintain a stable state over 30 days (35), we observed significant infection-induced alterations (ANOVA, *P* < 0.05), leading to higher variations in the categories between the sampling days. For example, the categories “translation, ribosomal structure and biogenesis,” “energy production and conversion,” and “carbohydrate transport and metabolism” showed greater variance at the earlier time points day 0, day 4, and day 7 after first H1N1 infection, while these categories were stable in the healthy animals. Moreover, an increased expression of proteins assigned to the functional categories “amino acid transport and metabolism” and “lipid transport and metabolism” was detected, while the abundance of PGs assigned to “cell motility” and “posttranslational modifications, protein turnover, chaperones” decreased on days 21 and 25 in the influenza-infected swine (Fig. 5). A detailed list of PGs showing varying abundance within the eggNOG categories is in the appendix (Table S7). Furthermore, analysis of the development of the functional composition over time showed that the functional categories “amino acid transport and metabolism,” “energy production and conversion,” “lipid transport and metabolism,” and “nucleotide transport and metabolism” were assigned to a common cluster. Subsequently, the corresponding individual sampling days of the two conditions were compared using the robust edgeR pipeline (FDR = 0.05) to identify significant changes within the functional categories between healthy and infected specimens. At day 0, we identified a significantly increased relative abundance of PGs assigned to subcategory “defense mechanisms” (FDR = 0.0003) in the influenza-infected swine. At day 4, most categories were found at the same state as that of the control. Significant changes were detected in the relatively low abundant categories “replication, recombination and repair” (FDR = 0.003) and “cytoskeleton” (FDR = 0.002). Comparing functional assignments from day 7 of the healthy and infected swine revealed no significant changes. This might indicate a process of recovery from the first H1N1 infection. Even though second IAV infection had only minor influence on the immune response of the animals (31), we detected significant changes in the functional composition of the microbiome at the corresponding day 21. For instance, relative abundances of PGs assigned to the categories “carbohydrate transport and metabolism” (FDR = 0.04) and “lipid transport and metabolism” (FDR = 0.002) were significantly increased, while decreased expression of proteins assigned to the categories “posttranslational modification, protein turnover, chaperones” (FDR = 0.006) and “cell motility” (FDR = 0.001) was observed. Furthermore, we detected significant increased abundances in the categories “amino acid transport and metabolism” (FDR = 0.01) as well as significant decreased amounts of PGs assigned to the category “cell motility” (FDR = 0.00004) at day 25 (Fig. 5; Fig. S6). The category “cell motility” was decreased in relative abundance mainly due to reduced amounts of flagellin from the families *Clostridiaceae* and *Lachnospiraceae*. Studies on inflammatory bowel disease (IBD) described a production of antibodies targeting flagellins of commensal bacteria, as flagellins were dominant antigens (58, 59). A similar observation of correlating reduced flagellin expression and decreased abundances of *Clostridiaceae* was made in the murine model during the course of acute colitis (60). Schwab and colleagues observed a correlation between the abundance of flagellin transcripts and the expression of TLR5, which has a regulatory effect on the bacterial load and species composition of *Firmicutes* and *Bacteroidetes* (60, 61). A response toward flagellins could be a possible explanation for the observed reduced abundance of *Clostridiaceae* and *Lachnospiraceae* and the altered *Firmicutes*/*Bacteroidetes* ratio.

**TABLE 2:** Assignment of identified PGs from feces of healthy (*n* = 3) and IAV-infected (*n* = 3) swine to functional categories (based eggNOG database); data are shown as mean value for all sampling days with the corresponding standard deviation

Category	Relative abundance (%)			
	Avg. healthy	SD	Avg. infected	SD
Translation, ribosomal structure, and biogenesis	43.4	0.7	43.9	7.9
Energy production and conversion	9	0.2	9.3	1.7
Carbohydrate transport and metabolism	7.8	0.7	9.4	2.2
Posttranslational modification, protein turnover, and chaperones	6.5	0.7	4.5	0.6
Cell motility	4.4	1.1	3.1	0.4
Amino acid transport and metabolism	3.6	0.3	4.5	1.4
Cell wall/membrane/envelope biogenesis	3.1	0.3	3.1	0.4
Inorganic ion transport and metabolism	2.6	0.4	3.1	0.8
Lipid transport and metabolism	1.7	0.1	2.1	0.7
Transcription	1.5	0.2	1.3	0.2

**FIG 6:**
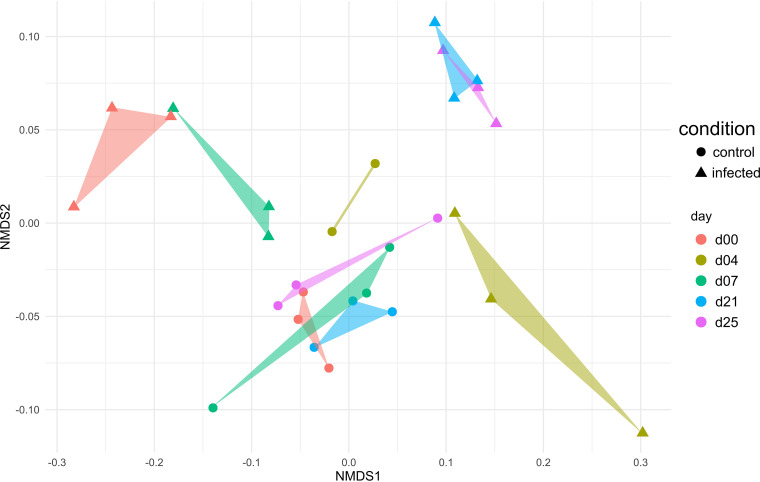
NMDS plot, based on Bray-Curtis dissimilarities, illustrating the temporal succession within the functional composition of the fecal microbiome in healthy and IAV-infected swine over 25 days. The classification of protein groups was based on their subrole according to the eggNOG database. Circles represent healthy swine and triangles represent infected swine.

**FIG 7:**
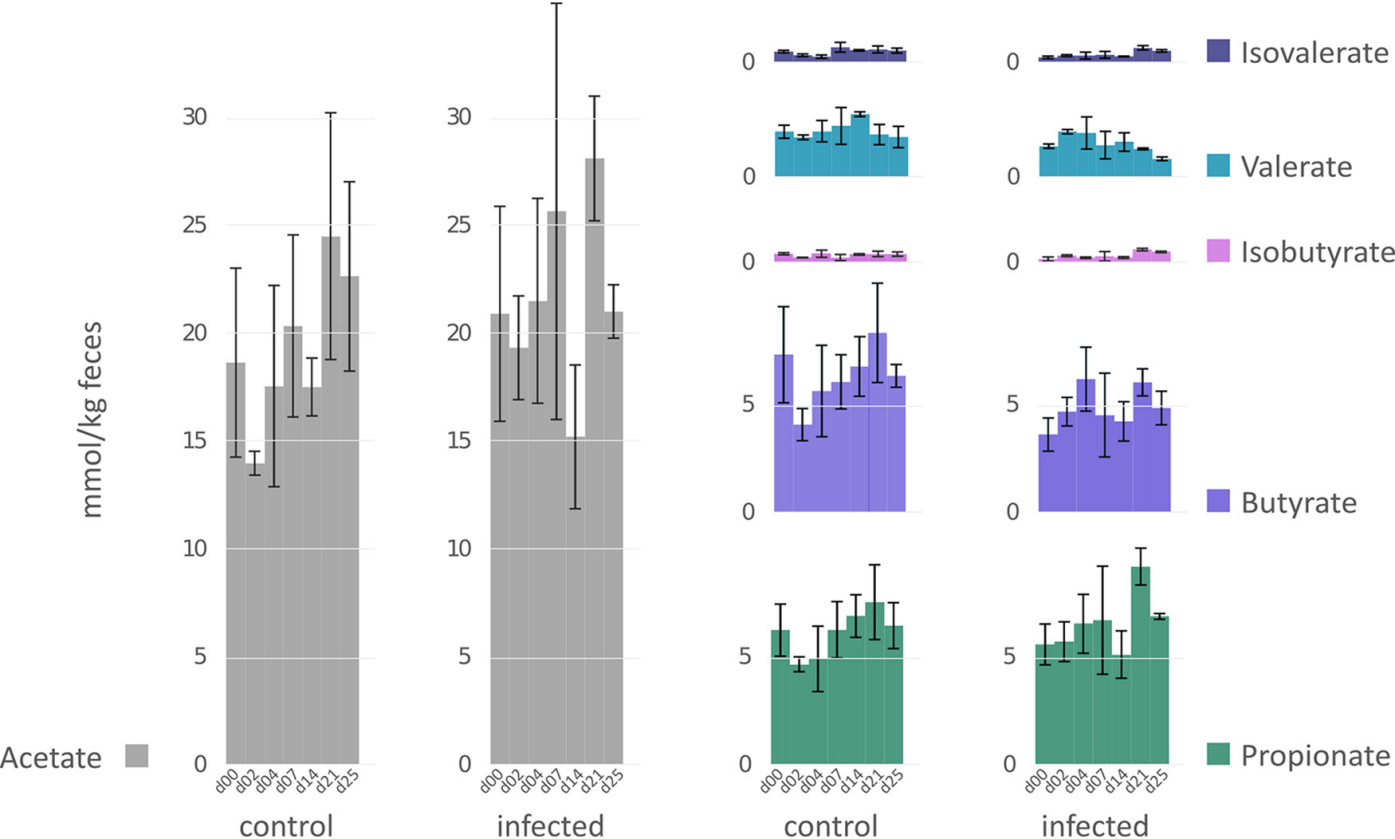
SCFA profile of healthy and influenza A-infected swine over a time period of 25 days. *y* axes: SCFA concentrations in mmol/kg feces; *x* axes: sampling day; all axes were in the same scale.

The influence of IAV infection on the functional composition of the fecal microbiome was further characterized using NMDS plot analysis. While the clusters were in close proximity to each other under healthy conditions, the viral infection led to a disturbance of this stable state. The distribution of the sample-specific clusters of the infected animals varies, particularly at the early sampling days (day 0, day 4, and day 7), and appeared to be stabilized at later time points after the second infection (day 21 and day 25) (Fig. 6). These findings correlated with observations of Schwaiger and colleagues, who described that the first IAV infection induced variations in blood immune cells, whereas these were stable after the second infection (31). While functional redundancy has been observed in the composition of the fecal microbiome under healthy conditions (35, 57), the results of our study suggest that the robust composition at the functional level has been disrupted by the IAV infection, indicating a systemic effect on the porcine gastrointestinal microbiome. A recent study in the murine model demonstrated not only changes in the taxonomic composition of the gastrointestinal microbiome but also a decreased formation of short-chain fatty acids after sublethal influenza infection (30). SCFAs are the major products of microbial fermentative activity in the gut resulting from the digestion of dietary fibers, resistant starch, proteins, and peptides (62, 63). In addition to their function as an energy source, SCFAs are known to improve the health status of the hosts. For instance, SCFAs were linked to the control of appetite/food intake (64), protective properties against colorectal cancer and inflammation in the gut (65, 66), and alteration of cell proliferation, apoptosis, and differentiation and their importance in the hosts’ immune response (67). In the present study, metabolome analysis revealed a similar temporal succession of SCFAs over 25 days in healthy and IAV-infected swine (Fig. 7). Significantly increased levels of acetate (Welsh *t* test, *P* = 0.02) were measured at day 2 after the first H1N1 infection. Acetate might have a supportive function in the recovery of infected animals, as acetate supplementation reduced lethal outcome of superinfections in the murine model (30). Further, our metabolome analyses detected significantly increased levels of propionate (*P* = 0.02) and isobutyrate (*P* = 0.01) at day 21 (Fig. 7), which was in good accordance with the increased expression of proteins involved in SCFA production, i.e., acetate kinase, phosphate acetyltransferase, acyl coenzyme A (acyl-CoA) dehydrogenase, and/or methylmalonyl-CoA mutase at later days of the infection (Table 3). In addition to the increased relative abundance of enzymes involved in the formation of SCFAs, increased abundances of *Prevotellaceae* and *Lachnospiraceae* were detected at days 21 and 25. These taxa have the potential to produce SCFAs in the gut (67). It is known that in the absence of easily fermentable fibers, SCFA are synthesized from less favorable sources, e.g., dietary amino acids or fats (68, 69). This could explain the increased abundance of PGs assigned to the categories “amino acid transport and metabolism” and the higher expression of proteins involved in SCFA synthesis, as observed at days 21 and 25 after infection, resulting in only minor alterations of the total SCFA concentration at the corresponding time points (Table 3; Fig. 5 and 7). Therefore, we speculated that IAV infection led to reduced food intake, and SCFAs upon infection are synthesized from dietary fibers and, additionally, from less-favorable amino acids.

**TABLE 3:**
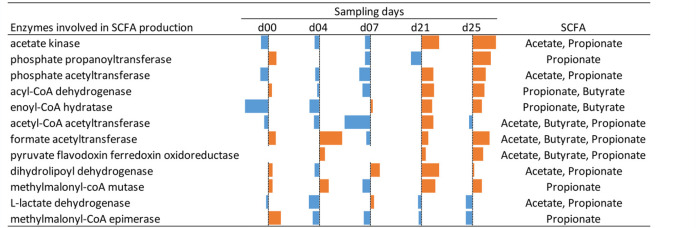
Occurrence of enzymes involved in SCFA production in the microbiome of IAV-infected animals, compared to their abundance in the healthy porcine microbiome^*a*^

a Bars represent log_2_ transformed fold changes (infection/control) at the corresponding sampling day. Increased amounts are shown as orange bars and decreased levels as blue bars.

### Conclusion.

In this study, we were able to characterize the influence of an IAV H1N1pdm09 infection on the respiratory and gastrointestinal microbiome of swine. We showed that IAV infection significantly alters the taxonomic composition of the upper respiratory tract and gastrointestinal microbiome. Comparing the URT microbiomes of healthy and infected animals revealed changes in the relative abundances of the predominant *Moraxellaceae* and *Pasteurellaceae*. Surprisingly, the observed longitudinal development of the respiratory tract microbiome structure was unaffected by the viral infection.

In contrast, our multi-omics analysis of swine feces showed that the microbial composition of the gastrointestinal microbiome was clearly affected by the IAV infection. We found increased microbial richness and diversity in the gastrointestinal microbiome and a disturbance of the longitudinal development during viral infection, as demonstrated by NMDS plot analysis. Exemplary for these alterations was the significant disturbance in the longitudinal development of *Lactobacillaceae*, as well as the absence of *Streptococcaceae* at the end of the experiment. Therefore, reduced abundance of *Streptococcaceae* could be a possible indicator of IAV infection in the pig. Moreover, metaproteome analysis revealed that the stable functional composition of the healthy microbiome can be significantly altered due to IAV infection. For example, proteins involved in SCFA production were affected by the viral infection. This finding was confirmed by metabolome analysis, measuring slightly induced concentrations of propionate and isobutyrate in feces after IAV infection. Since a faster recovery from second viral infection had been described, this possible correlation is an intriguing starting point for further studies.

## MATERIALS AND METHODS

### Animal study design.

**FIG 8:**
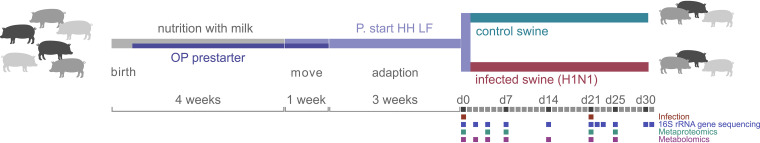
Experimental setup and sampling scheme of the IAV H1N1pdm09 infection trial in German Landrace pigs. Animals were fed OlymPig prestarter diet (OP prestarter) (Agravis, Münster, Germany) in addition to mother’s milk from day 3 after birth for 4 weeks. After the pigs were weaned, they received a mixture of OlymPig prestarter and PANTO start (P. start HH LF) (Hamburger Leistungsfutter GmbH, Hamburg, Germany) diet for 1 week. Afterwards, PANTO start diet was fed to the animals only. At the age of 8 weeks, the animal trial started. Days of viral infection (day 0 and day 21) are marked by red squares. Samples were taken over 25 days after the first infection. Individual sampling days for 16S rRNA gene sequencing, metaproteomics, and metabolomics are illustrated in blue, cyan, and purple squares, respectively.

The samples analyzed in this study were part of the animal trial approved by the State Office for Agriculture, Food Safety and Fishery in Mecklenburg-Western Pomerania with the reference number 7221.3-1-035/17 and were provided by the Department of Experimental Animal Facilities and Biorisk Management of the Friedrich-Loeffler-Institut at Insel Riems (FLI) (31). All swine were fed with OlymPig prestarter (Agravis, Münster, Germany) diet in addition to mother’s milk from the third day after birth. At 4 weeks of age, pigs were transported from the breeder to the FLI BSL-2 facility, where they were housed in 3 groups, which were randomly divided into cohorts with equal numbers of males and females 2 weeks before the infection experiment started. Pigs received a mixture of the OlymPig prestarter and PANTO start (Hamburger Leistungsfutter GmbH, Hamburg, Germany) diet for 1 week after they arrived at the FLI. Subsequently, pigs were fed with the PANTO start diet only until the end of the experiment. OlymPig prestarter and PANTO start are both based on wheat, barley, and soybean. At the age of 8 weeks, animals were infected intranasally with 2 ml of influenza A H1N1pdm09 suspension (10^6^ 50% tissue culture infective dose [TCID_50_]/ml) (day 0) followed by a second IAV infection 21 days later. Individual fecal samples and nasal swabs from healthy (*n* = 3) and infected (*n* = 3) German Landrace pigs were analyzed to examine the longitudinal development of the respiratory and gastrointestinal microbiome over a period of 31 days. The experimental setup is illustrated in Figure 8 and the sampling scheme is shown in Table 4. After sampling, all samples were immediately frozen on dry ice and subsequently stored at –80°C (35).

**TABLE 4:** Sampling scheme for multi-omics analysis of swine feces from IAV H1N1pdm09-infected animals, including number of individual samples at the corresponding sampling day

Sample type	Analysis	No. of samples analyzed after day:										
		0	2	4	7	14	21	22	23	25	30	31
Nasal swabs	16S rRNA gene analysis	3	3	6	6	3	6	3	3	6	3	3
Fecal samples after homogenization	16S rRNA gene analysis	3	3	3	3	3	3	3	3	3		
	Metaproteomics	3		3	3		3			3		
	Metabolomics	4	4	4	4	4	3			3		

### Sample processing.

Fecal samples were processed as described previously (35). Briefly, 1 g of frozen feces was separated from the original sample by a sterile scalpel and placed in a Covaris tissue tube TT1. Afterwards, mechanical homogenization to fecal powder, with impact level 5, was performed by the Covaris CP02 CryoPrep (Covaris Ltd., Brighton, UK). The powder was aliquoted to 100 mg for each of the multi-omics analysis.

### 16S rRNA gene sequencing and bioinformatic processing.

Nucleic acids of individual fecal samples were extracted from 100 mg material using a bead beating phenol-chloroform protocol (70), followed by nucleic acid precipitation with 3 M Na-acetate and isopropanol. After washing with 70% vol/vol ethanol, the resulting DNA pellets were resuspended in diethyl pyrocarbonate (DEPC)-treated MilliQ water for downstream applications. DNA of nasal swabs was extracted via Qiagen power soil kit (Qiagen, Hilden, Germany) according to the manufacturer’s protocol. Bead beating was performed using the FastPrep - 24 5G instrument (MP Biomedicals, Santa Ana, USA) for 45 s at intensity 5.5 m/s. Subsequently, Qubit dsDNA broad range assay kit (Invitrogen) was used to quantify the DNA content (on average 5,623.8 ng [±479.8] DNA/nasal swab). For amplicon and index-PCR, using the V4 primer pair 515F (5′-GTG-YCA-GCM-GCC-GCG-GTA-A-3′)/806R (5′-GGA-CTA-CNV-GGG-TWT-CTA-AT-3′) with subsequent PCR cleanup between and after both amplifications with AMPure XP beads, the DNA was diluted to 5 ng/μl (71, 72). Quantification of the libraries was done via Invitrogen Qubit dsDNA broad range assay kit, normalized to a final concentration of 5 pM, denatured with NaOH, and sequenced by Illumina MiSeq with an approximate output from 10.000 to 370.000 reads per sample.

### Protein extraction, mass spectrometry, database assembly, and data analysis.

Metaproteomic analysis was performed as described previously (35). Briefly, a TRIzol-based extraction protocol was applied to approximately 100 mg fecal powder for protein extraction. Protein concentrations of the obtained protein extracts were measured by Pierce BCA protein assay (73–76). Subsequently, 30 μg protein was loaded on a 4 to 22% Criterion TGX precast gel (BioRAD, Hercules, CA, USA), stained with Colloidal Coomassie brilliant blue G-250 (77), and each lane was cut in 10 pieces. Before tryptic digestion, each piece was processed to smaller blocks and destained. Afterwards, ZipTip purification (C_18_, Merck Millipore, Billerica, MA, USA), according to the manufacturer’s protocol, was performed to desalt the peptide-containing solution. The purified peptide mixture was eluted in glass vials, and vacuum centrifugation was performed until the mixture was dry. The peptides were finally resuspended in 10 μl of 0.1% (vol/vol) formic acid for mass spectrometric analysis. Mass spectrometry was also performed as described previously (35). Proteins were identified by Mascot Daemon version 2.6.2 (Matrix Science Ltd., London, UK) against a custom database based on the results of 16S rRNA gene sequencing of the same fecal samples, followed by validation with Scaffold 4.8.7 and X!Tandem (version X!Tandem Alanine [2017.2.1.4]) (35). For taxonomic and functional protein analysis, the metaproteome annotation pipeline Prophane was employed (78) (https://prophane.de). Prophane used diamond blast combined with the NCBI nr protein database (version 08.08.2018) for taxonomic classification (parameters: E value 0.01, query-coverage 0.9, max-target-seqs 1). Hmmscan algorithm combined with eggNOG database (version 4.5.1) was applied for functional annotation (E value 0.01). For statistical analysis, ANOVA and Welsh *t* test were performed (*P* = 0.05) using MeV (55). NMDS plots, based on Bray-Curtis dissimilarities, were done in RStudio, using the metaMDS function from the “vegan” package, followed by visualization with the package “ggplot2” (79). The data set of the infected swine was compared to reanalyzed samples of healthy swine from the same experiment, previously published by Gierse et al. (35). The mass spectrometry proteomics data have been deposited to the ProteomeXchange Consortium via the PRIDE (80) partner repository with the data set identifier PXD020775 (healthy swine) and PXD024077 (infected swine).

For statistical data evaluation of differences between healthy and infected animals at the same sampling day, we employed the edgeR v. 3.32.1 (81, 82) package in R v. 4.0.4 (79) for analyzing the spectral count data. In edgeR, data analysis is based on overdispersed Poisson models, where differentially abundant proteins are detected with an overdispersion-adapted Fisher’s exact test analog (81). A false-discovery rate (FDR) of 0.05 to define significant differences was employed. For analyzing the data as time series, i.e., changes across time in the same condition, we used the Short Time Series Expression Miner (STEM) (83) for which data were imputed using the package zCompositions v. 1.3.4 (84) and centered log-ratio (clr) transformed employing the compositions v. 2.0-1 (https://CRAN.R-project.org/package=compositions) package in R. Data were clustered with the STEM clustering methods, with replicates set to be from different time points, the minimum correlation between repeats and the absolute expression change set to 0.5, all permutations used, and the FDR set to 0.05. All other settings were left at default values. In addition, we employed the R packages dplyr v. 1.0.5, tidyr v. 1.1.3, and data.table v. 1.14.0 for data preparation (https://cran.r-project.org/web/packages/tidyr/index.html; https://cran.r-project.org/web/packages/dplyr/index.html; https://cran.r-project.org/web/packages/data.table/index.html).

### Analysis of metabolites.

Metabolites were extracted as described previously (35). Briefly, 100 mg of fecal powder was disrupted with the specialized lysing matrix E (MP Biomedicals, Eschwege, Germany) by FastPrep treatment. The supernatant was transferred to a new tube and the FastPrep treatment was repeated once with 2 ml ice-cold water and 500 μl dichloromethane and once with 2 ml ice-cold water. Afterwards, supernatants were combined, vortexed, stored on ice, and finally centrifuged. Finally, the water/methanol phase was lyophilized. Samples for ^1^H-NMR analysis were resuspended in phosphate-buffered saline (PBS) and vortexed, and the supernatant was measured with a Bruker Advance II 600 NMR spectrometer (85). As described previously for the 16S rRNA gene sequencing and metaproteome analyses, the results of metabolomics were also compared with the findings of the study by Gierse et al. (35).

### Data availability.

Received sequences were submitted to European Nucleotide Archive (ENA), with the project number PRJEB42450, accession number ERP126308. All 16S rRNA gene sequencing data were processed with the dada2 pipeline-package version 1.11.1 (R-version 3.6.1) in R (79). Amplicon sequence variants (ASVs) were inferred, and representative sequences of ASVs were assigned to taxonomies against SILVA 132 database (86) after removing chimeras. R packages “vegan,” “ggplot,” “phyloseq,” plyr,” and “reshape2” were used for advanced bioinformatic processing (e.g., alpha and beta diversity). Subsequently, generated data of IAV-infected pigs were compared to a previously published data set of healthy animals from the same experiment by Gierse et al. (35).
