# Supplementary material for: Influenza A H1N1 Induced Disturbance of the Respiratory and Fecal Microbiome of German Landrace Pigs – a Multi-Omics Characterization
Source: Microbiol Spectr. 2021 Oct 6;9(2):e00182-21. doi: 10.1128/Spectrum.00182-21 (PMC8510242; doi:10.1128/Spectrum.00182-21)
Supplement: SUPPLEMENTAL FILE 1 — Supplemental material. Download SPECTRUM00182-21_Supp_1_seq10.docx, DOCX file, 1.0 MB. [file spectrum00182-21_supp_1_seq10.docx]

**Supplemental Material**

**Influenza A H1N1 induced disturbance of the respiratory and fecal microbiome of German landrace pigs - a multi-omics characterization**

**Laurin Christopher Gierse^1^, Alexander Meene^1^, Daniel Schultz^2^, Theresa Schwaiger^3^, Charlotte Schröder^3^, Pierre Mücke^1^, Daniela Zühlke^1^, Tjorven Hinzke^1,5^, Haitao Wang^1^, Karen Methling^2^, Bernd Kreikemeyer^4^, Jörg Bernhardt^1^, Dörte Becher^1^, Thomas C. Mettenleiter^3^, Michael Lalk^2^, Tim Urich^1^, and Katharina Riedel^1^***

^1^ Institute of Microbiology, University of Greifswald, Felix-Hausdorff-Str. 8, 17489 Greifswald, Germany; laurin.gierse@uni-greifswald.de (L.G.); alexander.meene@uni-greifswald.de (A.M.); pierre.muecke@uni-greifswald.de (P.M.); daniela.zuehlke@uni-greifswald.de (D.Z.); tjorven.hinzke@uni-greifswald.de (T.H.); joerg.bernhardt@uni-greifswald.de (J.B.); haitao.wang@uni-greifswald.de (H.W.) doerte.becher@uni-greifswald.de (D.B.); tim.urich@uni-greifswald.de (T.U.)

^2^ Institute of Biochemistry, University of Greifswald, Felix-Hausdorff-Str. 4, 17489 Greifswald, Germany; daniel.schultz@uni-greifswald.de (D.S.); methling@uni-greifswald.de (K.M.); lalk@uni-greifswald.de (M.L.)

^3^ Friedrich-Loeffler-Institut, Greifswald-Insel Riems, Südufer 10, 17493 Greifswald, Germany; thomasc.mettenleiter@fli.de (T.M.); theresa.schwaiger@boehringer-ingelheim.com (T.S.); charlotte.schroeder@fli.de (C.S.)

^4^ Institute for Medical Microbiology, Virology and Hygiene, Rostock University Medical Centre, Schillingallee 70, 18055 Rostock, Germany; bernd.kreikemeyer@med.uni-rostock.de

^5^ Institute of Marine Biotechnology e.V., Walther-Rathenau-Straße 49A, 17489 Greifswald, Germany; tjorven.hinzke@uni-greifswald.de(T.H.)

***** Correspondence: riedela@uni-greifswald.de (K.R.); Tel.: +493834-420-5900 (K.R.)


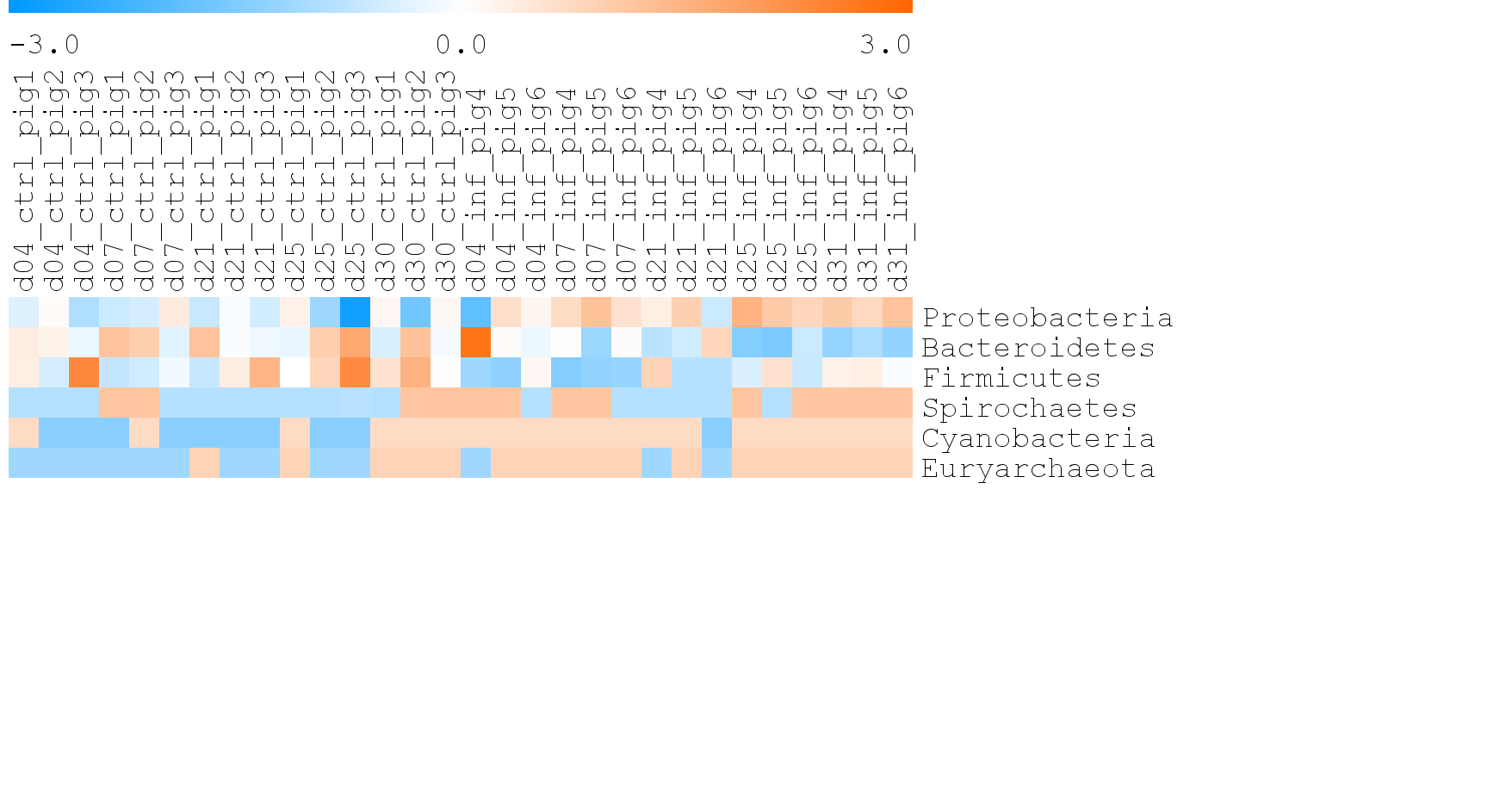


**Figure S1**: Heatmap of 16S rRNA gene sequencing data, showing detected phyla from the respiratory tract microbiome in pigs, that were significantly (ANOVA, p = 0.05) changed, due to Influenza A H1N1 infection. For statistical analysis and better illustration values were Z-transformed.


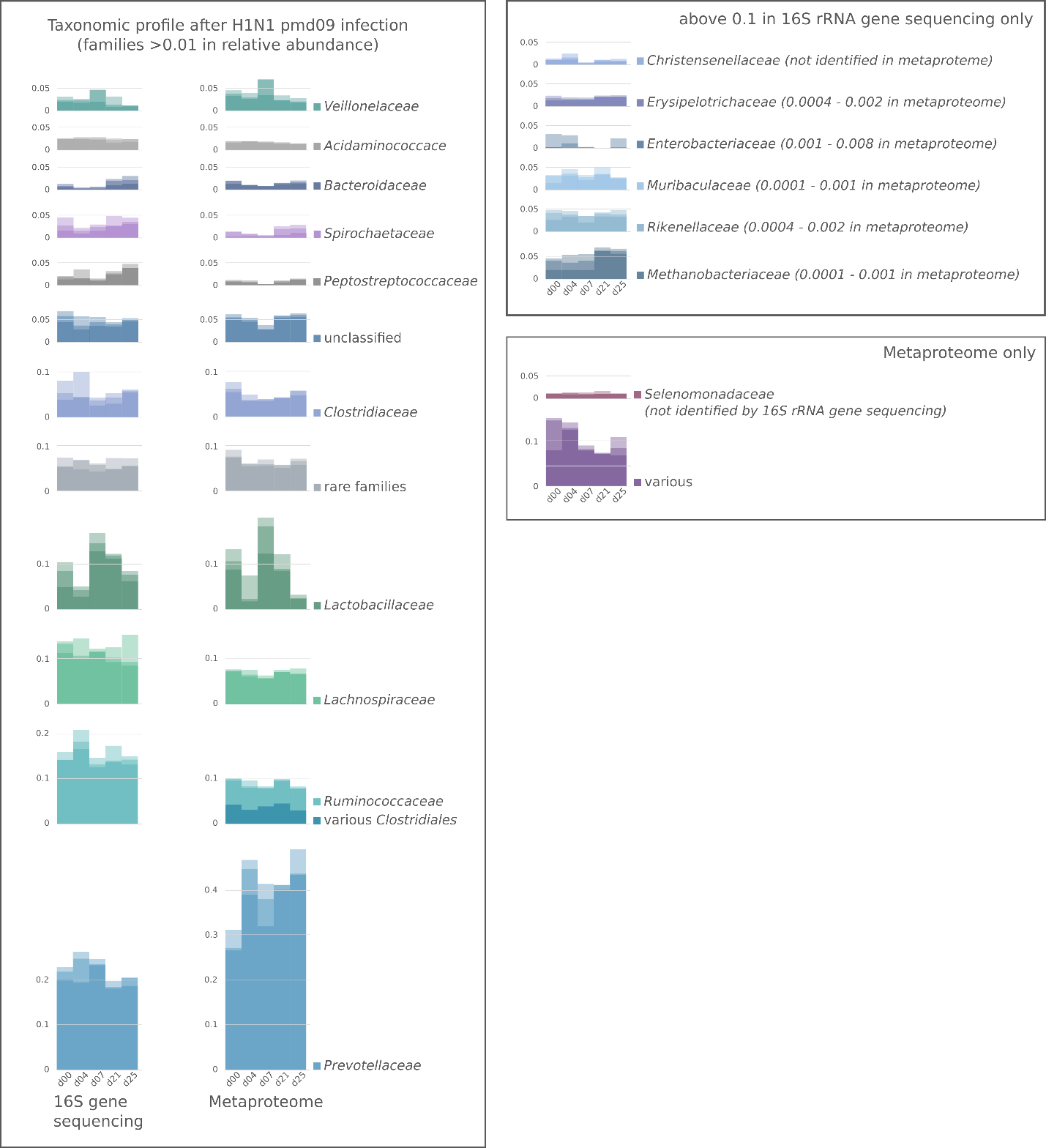


**Figure S2:** Comparison of the taxonomic profile from the gastrointestinal microbiome of IAV H1N1 infected pigs over a time period of 30 days, based on 16S rRNA gene sequencing and metaproteome analysis, using SILVA taxonomy. For better illustration, only families above 0.01 relative abundance were shown. X-axes: sampling day, Y-axes: relative abundance.


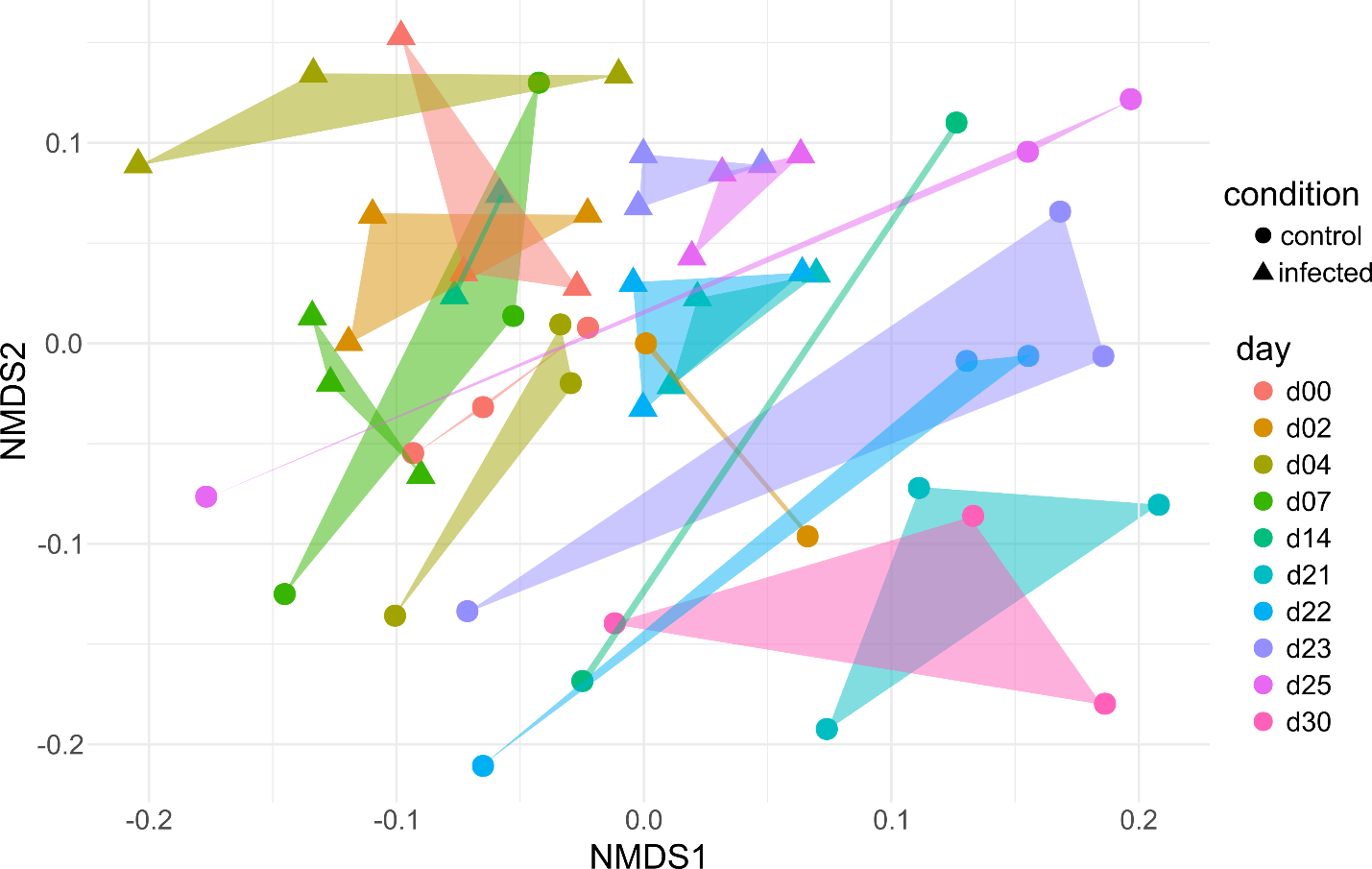


**Figure S3:** NMDS plot, based on Bray-Curtis dissimilarities, showing the composition of the fecal microbiome of healthy and IAV H1N1 infected pigs over a time period of 30 days.

**
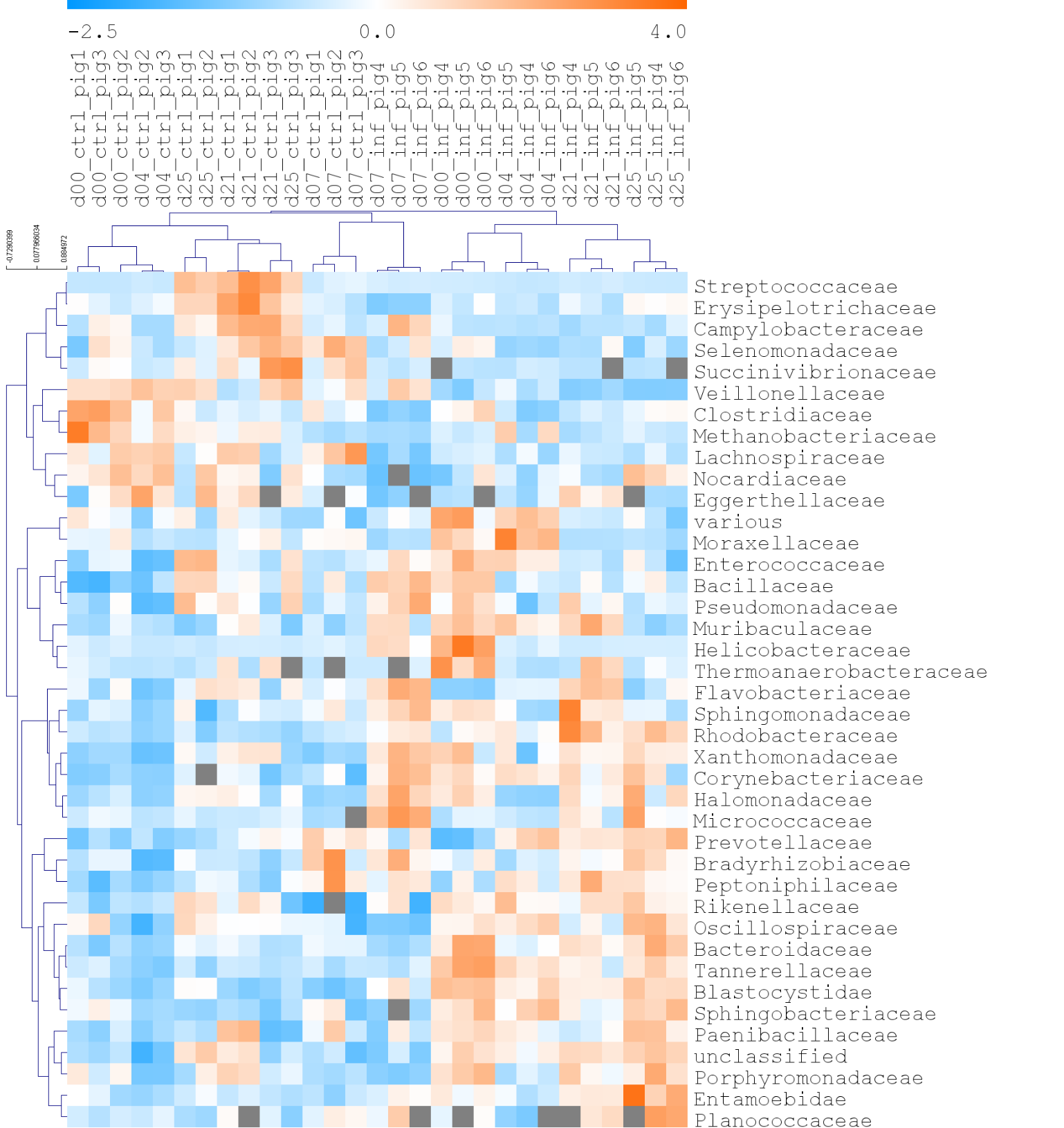
**

**Figure S4**: Hierarchical heatmap of the gastrointestinal microbiome of healthy and IAV infected pigs, based on Z- transformed metaproteome data. Only families that were significantly changes (ANOVA, p = 0.05) due to the infection were shown.

**
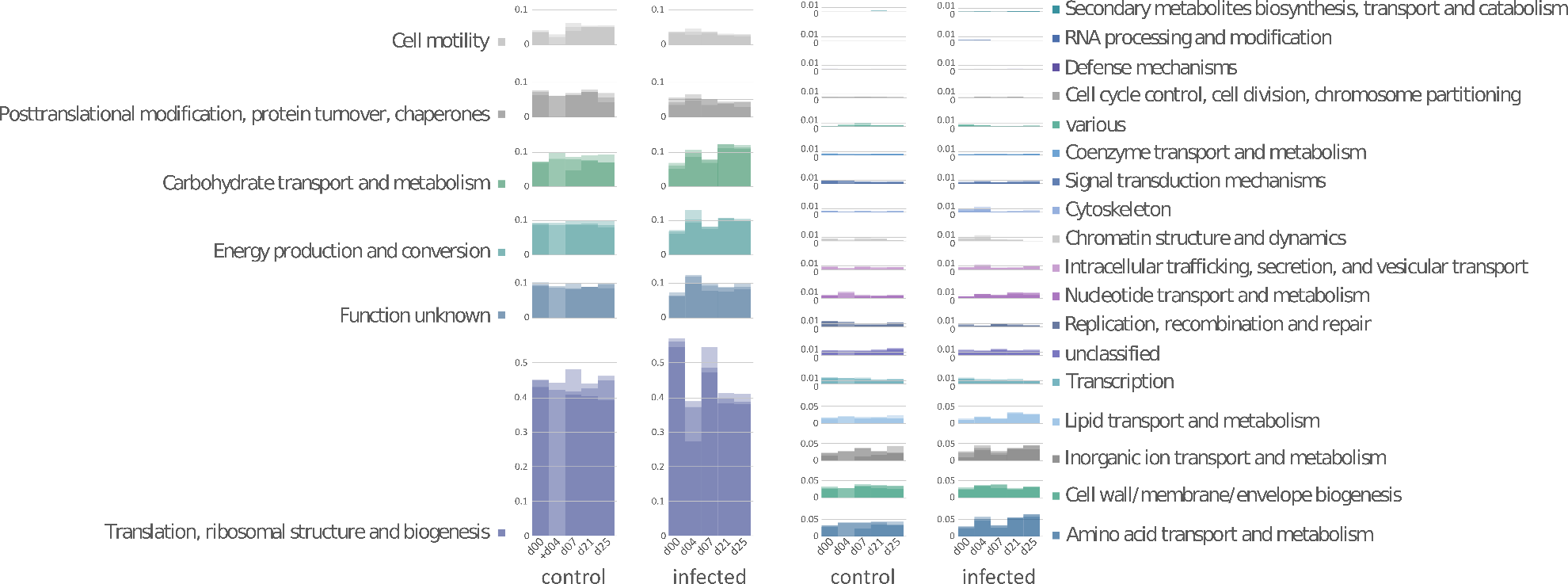
Figure S5:** Functional assignment (based on eggNOG database) of identified PGs from fecal samples of healthy (N = 3) and IAV-infected (N = 3) swine, collected over a period of 25 days. Individual bars were shown as overlay on the corresponding sampling day, representing individual swine. The darker the shade, the higher the accordance between the individual animals. X-axes: sampling day, Y-axes: relative abundance, +: one sample missing for the corresponding day.
